# The Proper Relation of the Superintendent to the Trustees of a Hospital
*Read at the twelfth annual meeting of the American Hospital Association, September 20 to 23, 1910.


**Published:** 1911-01-21

**Authors:** Henry M. Hurd

**Affiliations:** Superintendent, the Johns Hopkins Hospital, Baltimore, Md.


### SPECIAL INSTITUTIONAL ARTICLES.

### THE PROPER RELATION OF THE SUPERINTENDENT TO THE TRUSTEES OF A HOSPITAL.*

By HENRY M. HURD, M.D., Superintendent, the Johns Hopkins Hospital, Baltimore, Md.

This brief paper is the outcome of a conversation in a group of hospital officers in an eastern city a few months ago. In the course of this conversation an inquiry had been made as to whether or not in hospitals, as a rule, a superintendent was expected to be present at all general meetings of the governing body. This inquiry elicited varying replies. In one or more hospitals the superintendent, by virtue of the terms of his appointment, was required to be present; in other hospitals he might be present, but generally waited for an invitation to come; while in still other hospitals he was not expected to be present, and his presence was apparently not desired. I then stated that in my judgment it was desirable that the superintendent should be at meetings of the governing board, and finally consented to prepare a paper to present my personal view of the question.

### The Governing Body.

That the trustees of every hospital should be the governing body and that the superintendent should be the executive arm are self-evident truths to all who have had any experience in hospital work. The trustees are the fountain of all authority. To them are committed the custody of the funds, the management of the establishment, and the appointment of all employés and officers, professional, executive, and others, including the superintendent. The powers of the trustees or managers, however, are not executive and cannot be. They must work through others and set in order a machinery which will fulfil the object of the corporation. The trustees or managers of the hospital seem originally to have been a committee of the subscribers to the funds of the hospital, acting as an executive committee for the larger number who could not be convened oftener than once a year. The executive officer of such a committee was generally known as the secretary, and this name still persists in England. He was often a member of the board of trustees, and was especially delegated to undertake executive duties. A survival of this custom is found at the Government Hospital for the Insane at Washington, where a large board of visitors acts through a secretary, who performs all the duties of a superintendent and is the executive head of the hospital.

Under these various titles, whether secretary, superintendent, warden, or director, some person must be selected as the executive of every hospital. Hence it may be considered one of the most important functions of the trustees of a hospital to select a man to represent them. To paraphrase the language which I have known to be employed to define the duties of the president of a college, we may say the superintendent of a hospital "is the authorised means of communication between the trustees and the various officers and departments" of a hospital. It is his mission to carry out the policy of the managing board and to co-ordinate the efforts of every department. From the nature of things this board cannot attempt executive duties, but must act through officers of its own selection. Such chief executive officer, generally known as a superintendent, must consequently be responsible to the trustees for the welfare of the hospital and before the public. He has intimate relations with the medical staff, both resident and non-resident, and must know that they perform their duties faithfully, punctually, and efficiently. He has equally close relations with the economic staff, and must know that the housekeeping, laundry, kitchen, and other functions of a hospital are faithfully and efficiently performed. He must know about the nursing, the out-patient service, the purchase of food, clothing, fuel, and supplies. He is also interested in the erection and planning of buildings, the payment of bills, and the keeping of accounts.

### The Need for Co-operation.

In all of these varied duties there is absolute need of a close co-operation between the superintendent and the trustees or managing body. For this reason it is highly desirable that he should be present at meetings of the board of trustees to give information and assistance in attempting to solve the many perplexing problems which arise in hospital administration. The members of the board of trustees are generally busy men, who from the nature of their manifold employments may not be able to devote much thought to affairs of the hospital outside of the time employed in stated meetings and committee meetings. It is the business of the superintendent to keep himself conversant with every department of hospital work, and the information which he can give should be of material service to the board. It is consequently essential for many reasons that all meetings of trustees be held at the hospital buildings, so that the actual state of the hospital may be known at first-hand, and so that the knowledge of the superintendent may be utilised. The superintendent should be consulted in the appointment of subordinate officers. If possible he should recommend them, and the ultimate appointment should come from the board. He should have a voice in the appointment of house officers, especially where the hospital gives clinical facilities to a medical school. In many instances the superintendent has opportunities for becoming familiar with the character and conduct of these men which are denied to others. This knowledge should be utilised for the good of the service. He should have charge of discipline. I know a hospital which recently abolished a training school for nurses because it was thought to be an expensive adjunct, only to learn from the superintendent, who had not been consulted, that the action had materially increased the expenses of the hospital. I know another hospital in which the trustees took radical action in the matter of surgical fees and upset the equanimity of the whole surgical staff, only to learn that their action had been impracticable and unwise. The superintendent should have the confidence and cordial co-operation of every trustee. He is endeavouring to carry out their plans and to realise their wishes. Trustees and superintendents have the same objects in view. They should work in harmony, and each has a right to utilise the ability and experience of the other.

### The Need for Trained Men.

The time is past when a hospital superintendent can be satisfactorily selected apparently because he has made a failure in some other walk in life. The choice of one to perform the delicate and trying duties of the complex modern hospital can no longer be fortuitous. The increasing responsibilities of the position require that no mistakes be made, and that the man selected should be worthy to attain success by reason of ability and training. Formerly often a head clerk or accountant, he has now become by force of the circumstances of his position an expert in heating, ventilation, and plumbing, in hospital construction, in sanitary science, in hospital policies and administration, and in matters of education. He is often a physician, a clergyman, a trained business man, a teacher, an engineer; or she may be a physician or a trained nurse. He (or she) is no longer a young business man serving an apprenticeship, but a man or woman of mature judgment, broad experience, and ample training. It is to be regretted that as yet we have no systematic training of men and women to fill these positions in connection with existing institutions. This Hospital Association owes it to the country to arrange for courses in hospital administration to fit men and women for efficient service. Thus far we have all learned our duties by the force of hard circumstances in the emergencies of institution life, and too often at the expense of the institution. Would that some philanthropist could conceive of the importance of affording an adequate training for the large numbers of men and women who must be found to administer the rapidly growing hospital service of the country! The experience and knowledge of the superintendent should be utilised by the trustees of hospitals, and they cannot afford to deprive themselves of the presence of these men in their meetings. No large business can be successfully transacted by a board of trustees without the presence of the general manager. The more highly specialised and technical the work, the more necessary it is to have expert advice.

The superintendent should be given large powers and responsibilities, and should be upheld by the trustees when he is in the right, and held to a strict account when he is in the wrong.

* Read at the twelfth annual meeting of the American Hospital Association, September 20 to 23, 1910.

